# patcHwork: a user-friendly pH sensitivity analysis web server for protein sequences and structures

**DOI:** 10.1093/nar/gkac252

**Published:** 2022-04-19

**Authors:** Mirko Schmitz, Anne Schultze, Raimonds Vanags, Karsten Voigt, Barbara Di Ventura, Mehmet Ali Öztürk

**Affiliations:** Signaling Research Centres BIOSS and CIBSS, University of Freiburg, Schänzlestr. 18, 79104 Freiburg, Germany; Institute of Biology II, University of Freiburg, Schänzlestr. 1, 79104 Freiburg, Germany; Signaling Research Centres BIOSS and CIBSS, University of Freiburg, Schänzlestr. 18, 79104 Freiburg, Germany; Institute of Biology II, University of Freiburg, Schänzlestr. 1, 79104 Freiburg, Germany; Institute of Biology III, University of Freiburg, Schänzlestr. 1, 79104 Freiburg, Germany; Institute of Biology III, University of Freiburg, Schänzlestr. 1, 79104 Freiburg, Germany; Signaling Research Centres BIOSS and CIBSS, University of Freiburg, Schänzlestr. 18, 79104 Freiburg, Germany; Institute of Biology II, University of Freiburg, Schänzlestr. 1, 79104 Freiburg, Germany; Signaling Research Centres BIOSS and CIBSS, University of Freiburg, Schänzlestr. 18, 79104 Freiburg, Germany; Institute of Biology II, University of Freiburg, Schänzlestr. 1, 79104 Freiburg, Germany

## Abstract

pH regulates protein function and interactions by altering the charge of individual residues causing loss or gain of intramolecular noncovalent bonds, which may lead to structural rearrangements. While tools to analyze residue-specific charge distribution of proteins at a given pH exist, currently no tool is available to investigate noncovalent bond changes at two different pH values. To make protein pH sensitivity analysis more accessible, we developed patcHwork, a web server that combines the identification of amino acids undergoing a charge shift with the determination of affected noncovalent bonds at two user-defined pH values. At the sequence-only level, patcHwork applies the Henderson–Hasselbalch equation to determine pH-sensitive residues. When the 3D protein structure is available, patcHwork can be employed to gain mechanistic understanding of the effect of pH. This is achieved using the PDB2PQR and PROPKA tools and noncovalent bond determination algorithms. A user-friendly interface allows visualizing pH-sensitive residues, affected salt bridges, hydrogen bonds and aromatic (pi–pi and cation–pi) interactions. patcHwork can be used to identify patches, a new concept we propose of pH-sensitive residues in close proximity on the protein, which may have a major impact on function. We demonstrate the attractiveness of patcHwork studying experimentally investigated pH-sensitive proteins (https://patchwork.biologie.uni-freiburg.de/).

## Graphical Abstract

**Graphical Abstract:**
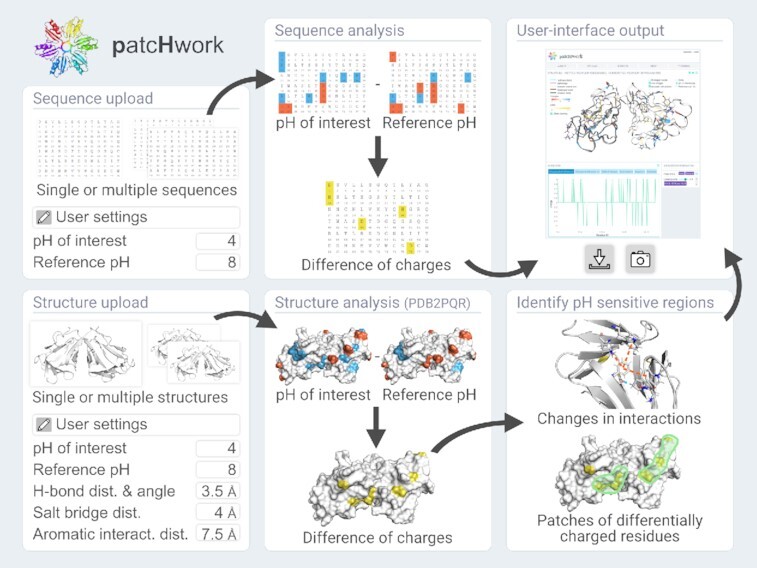
Demonstration of pH sensitivity analysis with patcHwork.

## INTRODUCTION

The concentration of hydrogen ions in a solution (used to calculate the pH) determines the charge of the side chains of the amino acids in a given protein by regulating their protonation state. Various properties such as protein solubility (1), stability (2), ability to interact with other molecules (3), flexibility (4) and activity (5) are affected by pH. As a matter of fact, the amount of protonation of the amino acid side chains in proteins has been proposed to be a new form of protein post-translational modification (6). Ionizable residues in proteins react to the surrounding pH according to their ionization constant (p*K*_a_) and they become positively or negatively charged at pH values below or above their p*K*_a_, respectively. This adaptation can cause loss or gain of intramolecular noncovalent bonds between residues, which can subsequently result in structural rearrangements that could regulate protein function and interactions (7,8). Thus, it is crucial to understand the effect of pH change on a protein structure and determine any resulting adjustments in intramolecular noncovalent bonds.

Currently, a number of web servers exist that allow calculating the charges of the amino acid side chains at a certain pH using either sequence or structural information (9–11). VOLPES (10), for instance, applies the Henderson–Hasselbalch equation (12) to calculate the charges of the side chains of the amino acids in a protein of interest at a user-defined pH using only sequence information. However, the Henderson–Hasselbalch equation does not account for the influence of neighboring amino acids on the p*K*_a_ of a residue side chain (13). An analysis at the level of the structure is, therefore, clearly required to precisely determine the effect of pH change on proteins.

Structure-based analysis of pH-mediated changes in the charge of the side chains of amino acids has benefited from datasets obtained from NMR experiments, which significantly helped refine computational methods for p*K*_a_ prediction of residues in protein structures (14). For example, APBS (11) calculates the electrostatic potentials of proteins by assigning charge and radius to atoms using the PDB2PQR (15) and PROPKA (16) tools. Protein-sol (9) provides overall pH-dependent charge information for proteins of interest, as well as predictions of the destabilization of residue-specific electrostatic interactions due to limited ionization ability of buried amino acids (13). There is also a graphical user interface (GUI) plug-in implementation (17) on the VMD software (18) to visualize PROPKA (16) predictions. While being useful, these approaches do not allow the user to easily monitor the appearance/disappearance of intramolecular noncovalent bonds (salt bridges, hydrogen bonds, pi–pi and cation–pi interactions) when the pH is changed from one value to another. On the other hand, these bonds can be investigated only at the default physiological pH with web servers such as RING 2.0 (19), Arpeggio (20) and ProteinTools (21). Taken together, currently no tool offers the possibility to directly observe protonation changes of amino acids caused by a shift in pH between two user-defined values and the resulting gain/loss of noncovalent bonds in the protein structure. Therefore, researchers wishing to analyze pH sensitivity of a given protein need to use several tools in parallel with manual curation of the outputs, which makes such an analysis difficult when the knowledge in computational structural biology is limited.

Here, we present patcHwork, a novel web server that supports high-throughput pH sensitivity analysis at two user-defined pH values at either the sequence or structure level. At the sequence level, patcHwork allows submitting up to 10 000 protein sequences, which are then analyzed using the Henderson–Hasselbalch equation (12) to determine pH-sensitive residues at the user-defined pH values. When the 3D protein structure is available, further mechanistic understanding of the effect of pH on a protein of interest can be obtained by the execution of the PDB2PQR (15) and PROPKA (16) software and noncovalent bond determination algorithms (22–25). pH-sensitive residues and pH-mediated changes in salt bridges, hydrogen bonds and aromatic (pi–pi and cation–pi) interactions are visualized in an interactive GUI. Additionally, users obtain information regarding so-called patches, groups of pH-sensitive residues found in a customizable physical distance on the protein structure, which may play a more profound role than individual amino acids in the regulation of protein function upon pH change. To demonstrate the workflow and the power of patcHwork, we carried out sequence-based pH sensitivity analysis of *Escherichia coli* cell envelope proteins, and structure-based analysis of the taste-modifying protein neoculin (26,27) and the pH-regulated mouse anion exchanger 2 (mAE2) protein (28–32).

## FUNCTIONALITIES OF patcHwork

patcHwork has four main computational components to investigate the response of proteins to a change in pH: protein sequence-based analysis, protein structure-based analysis, noncovalent bond analysis and identification of pH-sensitive patches.

### Protein sequence-based analysis

The Henderson–Hasselbalch equation (12) is solved for each amino acid of the submitted protein FASTA sequences at two user-given pH values. Residue-specific charges at each pH value and also delta charges are provided as an interactive output. In order to rank the proteins based on their pH sensitivity, an ‘overall charge score’ is defined as follows: for each protein, the sum of the charges at the pH of interest is subtracted by the sum of charges at the reference pH and then normalized by the total number of residues in the protein.

### Protein structure-based and noncovalent bond analyses

The protonation state of each amino acid in the submitted protein PDB structures is calculated using the PDB2PQR (15) and PROPKA (16) tools at the two user-given pH values. In addition to residue-specific charge information, created and destroyed noncovalent bonds (salt bridges, hydrogen bonds, and pi–pi and cation–pi interactions) upon pH change are determined and given as an interactive output.

### pH-sensitive patches

On protein structures, residues that change their protonation state at a given pH shift and are found within a radius of ≤8 Å from each other (customizable) are defined as a pH-sensitive patch.

Further details of sequence and structure-based analyses, pH-sensitive patch identification and noncovalent bond determination, as well as the web server implementation, are given in Supplementary Data.

## CASE STUDIES

### Sequence-based pH sensitivity analysis of *E. coli* cell envelope proteins

**Figure 1.**
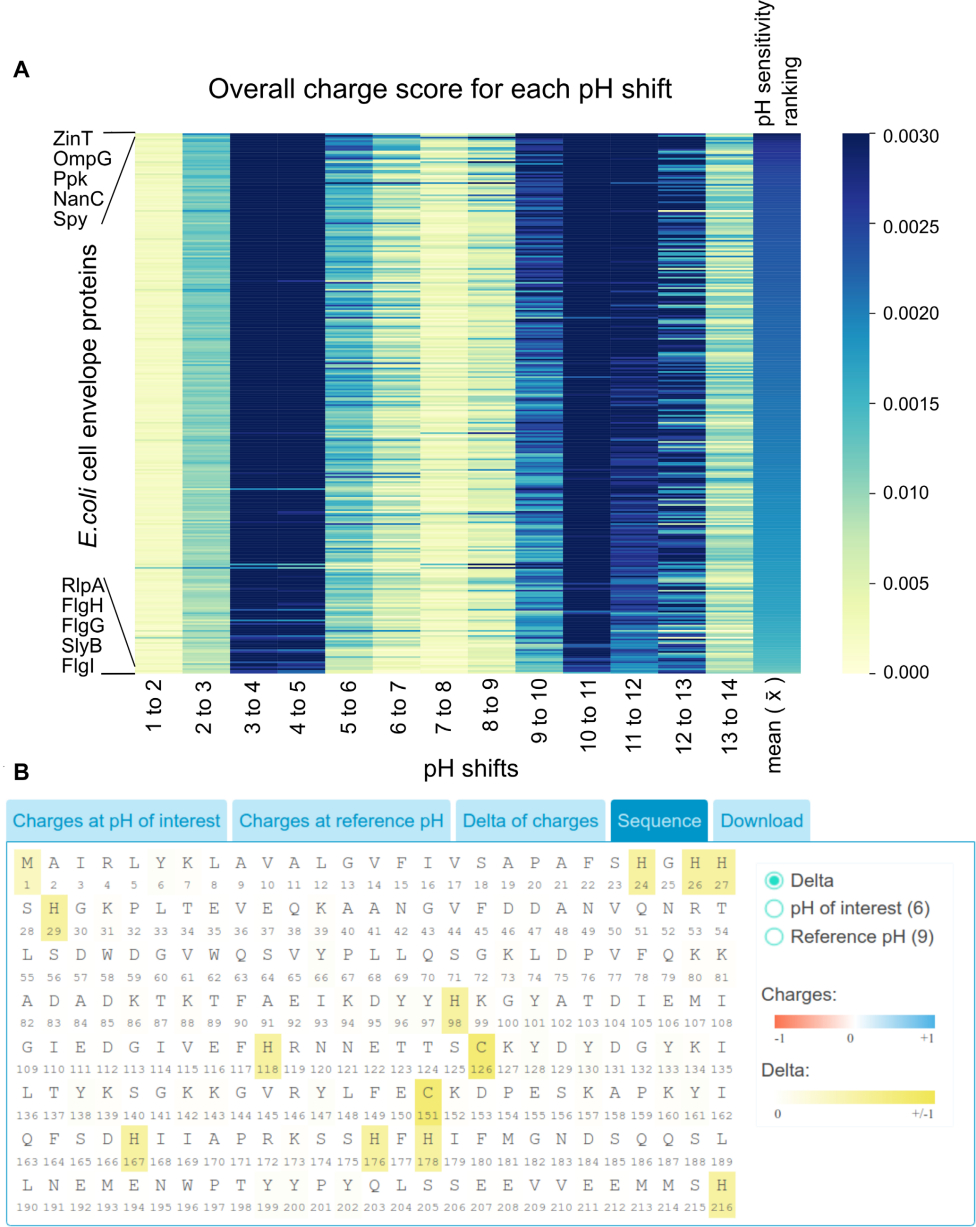
Demonstration of sequence functionality of patcHwork. (**A**) Overall ‘charge score’-based pH sensitivity ranking of 309 *E. coli* cell envelope proteins (GO:0030313) for one unit incremental pH increase from pH 1 to 14. $\overline{x}$ represents the mean of all overall charge scores for each protein. *Note*: Uncharacterized proteins are not considered in Supplementary Table S1. (**B**) ZinT, the most pH-responsive *E. coli* cell envelope protein from panel (A), is analyzed for the shift in pH from 6 to 9 with patcHwork using its sequence functionality.

To demonstrate the advantage of using patcHwork for pH sensitivity analysis, we asked whether we could identify in a high-throughput manner proteins that are mostly affected by pH looking at a family of proteins that is exposed to the extracellular medium and, consequently, is most likely affected by pH change than cytoplasmic proteins: cell envelope proteins. We collected the FASTA sequences of 309 *E. coli* proteins annotated as being part of the cell envelope (GO:0030313) and determined for each the overall charge score (residues’ total charge shifts normalized by the protein length) when changing the pH from 1 to 14 with an increment of 1 (i.e. pH of interest 2, reference pH 1; then, pH of interest 3, reference pH 2; and so on; for details see Supplementary Data). Next, we created for each protein a mean score ($\overline{x}$) taking the mean of the overall charge scores of all pH shifts (Figure 1A). We propose that, by ranking the proteins based on the $\overline{x}$ score, we are able to identify the most and least pH-responsive proteins, respectively. After obtaining this ranked list, we checked the literature for experimental information on the top and bottom five proteins to verify whether they are reported to be involved in pH regulation/responsiveness or not. Interestingly, while four out of the five top ranked proteins are reported to be pH-responsive/regulated, for the bottom five proteins only one is reported to be regulated by/sensitive to pH (see Supplementary Table S1). When we ran the top-ranked ZinT protein sequence in patcHwork at pH values of 6 and 9, we captured the amino acids involved in metal ion binding (residues 24–29 and 166–178), which have been previously implicated in pH sensing (Figure 1B) (33). This example illustrates how, using patcHwork, it is possible to conduct quantitative pH sensitivity analysis, ranking hundreds of protein sequences in a matter of minutes.

### Structural analysis of pH-induced changes in the sweet taste protein neoculin

To demonstrate how patcHwork can be used to narrow down potential residues likely to be involved in the pH response mechanism of a given protein, we analyzed neoculin, a heterodimeric protein from the plant *Curculigo latifolia*. Neoculin (also called curculin) consists of an acidic (NAS) and a basic (NBS) subunit and it turns sour into sweet taste (34). This effect has been shown to be induced by low pH (35). A mutant neoculin where all five histidines were mutated to alanine was shown to be active across pH conditions (26). Specifically, His11 in the NBS was identified as the main pH sensor responsible for the activity of the protein at low pH. Nakajima *et al.* suggested that low pH-mediated loss of the aromatic interaction between His11 and His14 in the NBS is essential for the pH-responsive function of neoculin (26).

**Figure 2.**
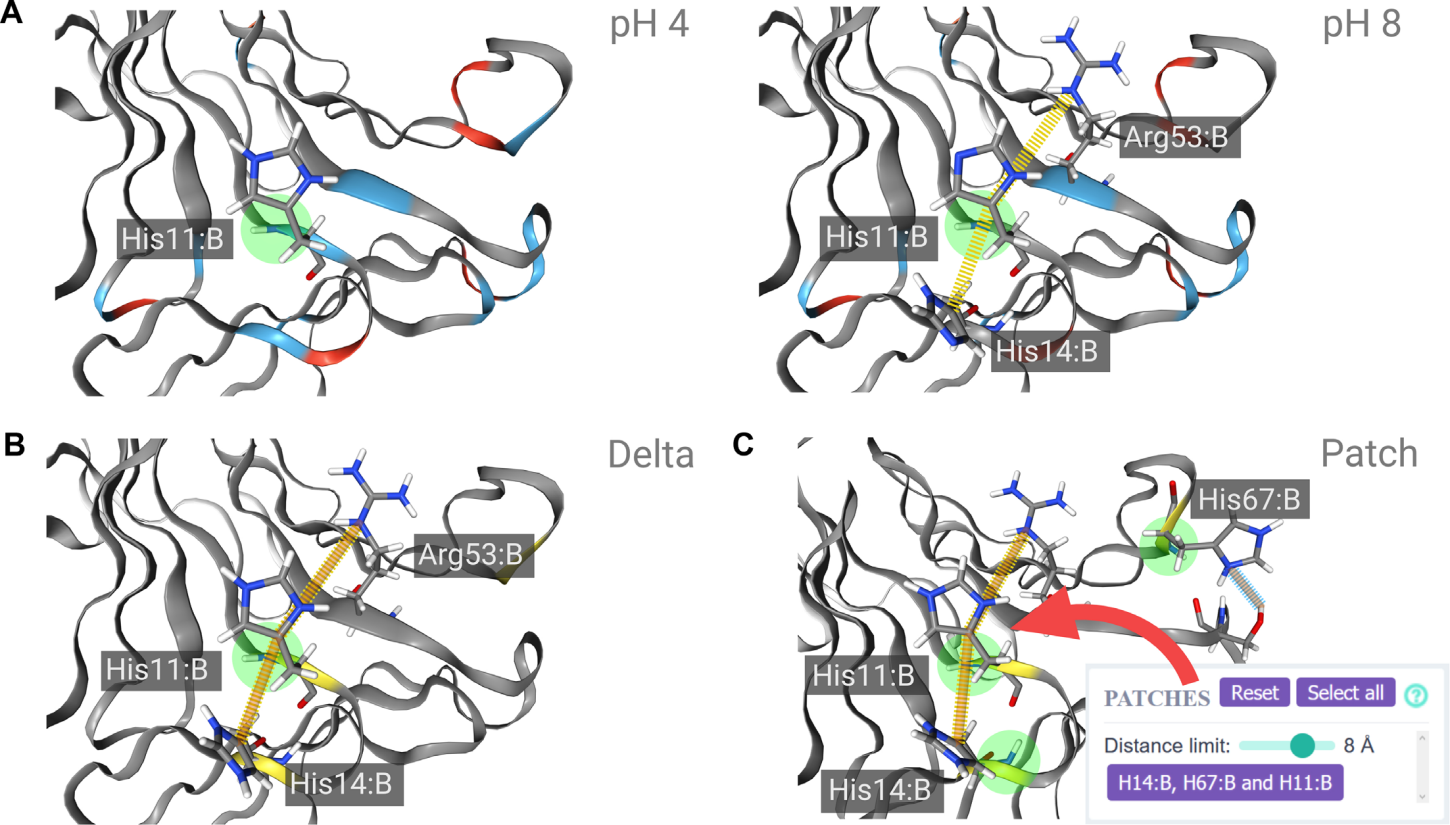
Structure-based patcHwork analysis using neoculin X-ray structure (PDB ID: 2D04, chains A and B) at pH values of 4 and 8. (**A**) The intramolecular pi–pi interaction between His11 and His14 is shown in yellow at pH 8. (**B**) Delta view in patcHwork. Intramolecular noncovalent bonds that are present only for one of the two pH values are shown in orange. (**C**) Patch view in patcHwork. The pH-sensitive patch, identified when the threshold for the distances between pH-sensitive residues is set to be ≤8 Å, is shown. Individual residues within the patch are displayed in the box. (A, B) The structure of neoculin with both its subunits (PDB ID: 2D04, chains A and B) was used in the analysis. The figure was generated in patcHwork with modifications to the residue labeling. Negatively charged residues are shown in red, positively charged residues in blue and neutral residues in white. Residues that are differentially charged at pH 8 and 4 are highlighted in yellow.

While it is intuitive to mutate histidine residues to investigate the pH response mechanism of a given protein considering that the p*K*_a_ of histidine is close to the physiological pH, analyzing the protein structure could give further valuable information to narrow candidate residues down as well as give insights into a potential mechanism. For this purpose, we ran a structure-based analysis of neoculin (PDB ID: 2D04, chains A and B) (36) with patcHwork for the pH shift from 4 to 8, the two extreme pH values used in the experimental study (26) (session can be accessed at https://patchwork.biologie.uni-freiburg.de/results.php?key=example_pdb). We found that the noncovalent bonds between Arg38 and His36 (aromatic interaction), His67 and Ser50 (hydrogen bond), Arg53 and His11, and His11 and His14 (aromatic interactions) are destroyed. Interestingly, His11 has two disrupted noncovalent bonds, while the other differentially charged residues only have single ones, hinting that a change in pH especially affects this region occurring in the Arg53–His11–His14 triad, which is in line with the experimental observations (26) (Figure 2A and B). In addition to the analysis of noncovalent bond changes, we identified a pH-sensitive patch constituted by His11, His14 and His67 (Figure 2C).

### Structural analysis of pH-regulated mAE2

To showcase the usefulness of patcHwork for the pH sensitivity analysis of proteins consisting of over a thousand amino acids, for which the shift in the p*K*_a_ of residues is due to the surrounding environment, we selected as the last case study an anion exchanger protein (also called bicarbonate transporter).

The anion exchangers 1–3 (AE1–3) mediate Na^+^-independent Cl^−^/HCO_3_^−^ exchange in several types of cells. Their functions range from gas transport to cell volume and intracellular pH regulation (37). Both AE1 and AE2 catalyze H^+^–SO_4_^2−^/Cl^−^ and H^+^–SO_4_^2–^/H^+^–SO_4_^2–^ exchange in a pH-sensitive fashion: in an acidic environment (pH of 5.5), the H^+^–SO_4_^2–^ efflux is maximal, whereas it sharply decreases at near-neutral pH (pH of 7.5) (28–31). In human AE1 (hAE1), the binding site for the proton that is cotransported with SO_4_^2–^ was proposed to be Glu681 (31). Chemically converting this negatively charged residue to an alcohol (Glu681OH), and thus rendering it neutrally charged, leads to proton-independent transport of SO_4_^2−^ as well as nearly complete elimination of the transport’s pH dependence (32,38). Mutating the corresponding conserved glutamic acid in mouse AE1(mAE1) and mAE2, Glu699 and Glu1007, respectively, to the neutral glutamine abolishes the preference of sulfate exchange in acidic medium similarly to the experiments with hAE1 (28–32,38).

In the absence of this experimental evidence, we would typically perform mutagenesis analysis on mAE2 to identify amino acids involved in the pH-regulated SO_4_^2–^ transport. To this aim, we would likely focus on the histidines, as their p*K*_a_ value is close to the physiological pH and thus we know that they would change protonation state for a pH shift from 5.5 to 7.5. This methodology would lead to identifying 35 candidate histidine residues in mAE2, which consists of 1237 amino acids. However, it would not lead to identifying Glu1007, which is reported to be the key pH-responsive amino acid of mAE2 (28–30).

Using patcHwork for the analysis allows avoiding falling in the prototypical histidine-oriented approach. As a matter of fact, by making use of p*K*_a_ predictions from PROPKA (16), patcHwork calculates the shift in pKa considering the surrounding environment of the side chains of each amino acid, since this is known to exert a role (13). We downloaded the mAE2 model structure from the AlphaFold Protein Structure Database (39,40) (model ID: AF-P13808-F1), removing regions with low model confidence score (<70) with the exception of the linker between the two domains (anion exchanger and cytoplasmic domains), and we submitted the structure to patcHwork using pH of interest of 5.5 and reference pH of 7.5, which were similarly used in the experimental studies (29,30). We obtained the following three levels of information (session can be accessed at https://patchwork.biologie.uni-freiburg.de/results.php?key=mAE2_example): (i) residues that change their protonation state (shown in yellow in patcHwork); (ii) residues that change their protonation state and cause noncovalent bond changes (yellow residues with orange and green stripes in patcHwork); and (iii) patches: residues that change their protonation state and are in close physical proximity with other residues that change their protonation state (green spheres in patcHwork). These three features generated from patcHwork can be used to identify pH-sensitive residues as seen in Table 1.

**Table 1.** Comparison of histidine-based and structure-based analyses (the latter performed with patcHwork) for the identification of pH-sensitive amino acids within mAE2

	Using mAE2 model structure in patcHwork for pH shift from 5.5 to 7.5		
Using mAE2 sequence and p*K*_a_ information for pH shift from 5.5 to 7.5	Protonation state shifting residues	At least one protonation state shifting residue + noncovalent bond change	Part of a patch (≤9 Å close to at least two other protonation state shifting residues)
35 histidines	14 histidines Glu338 Glu1007 Asp1031	Asp1031–Gln756 His1029–Asp684 Glu1007–Ser764 Asp684–His1029 His1145–His1160 His360–Arg570 Glu346–His360 His566–Arg570 Glu325–His566 Glu338–Arg341 His395–Arg412	[His425–His566–Glu338–His360] [Asp1031–His1029–Glu1007] [His1145–His1160–His1144–His1136]

**Figure 3.**
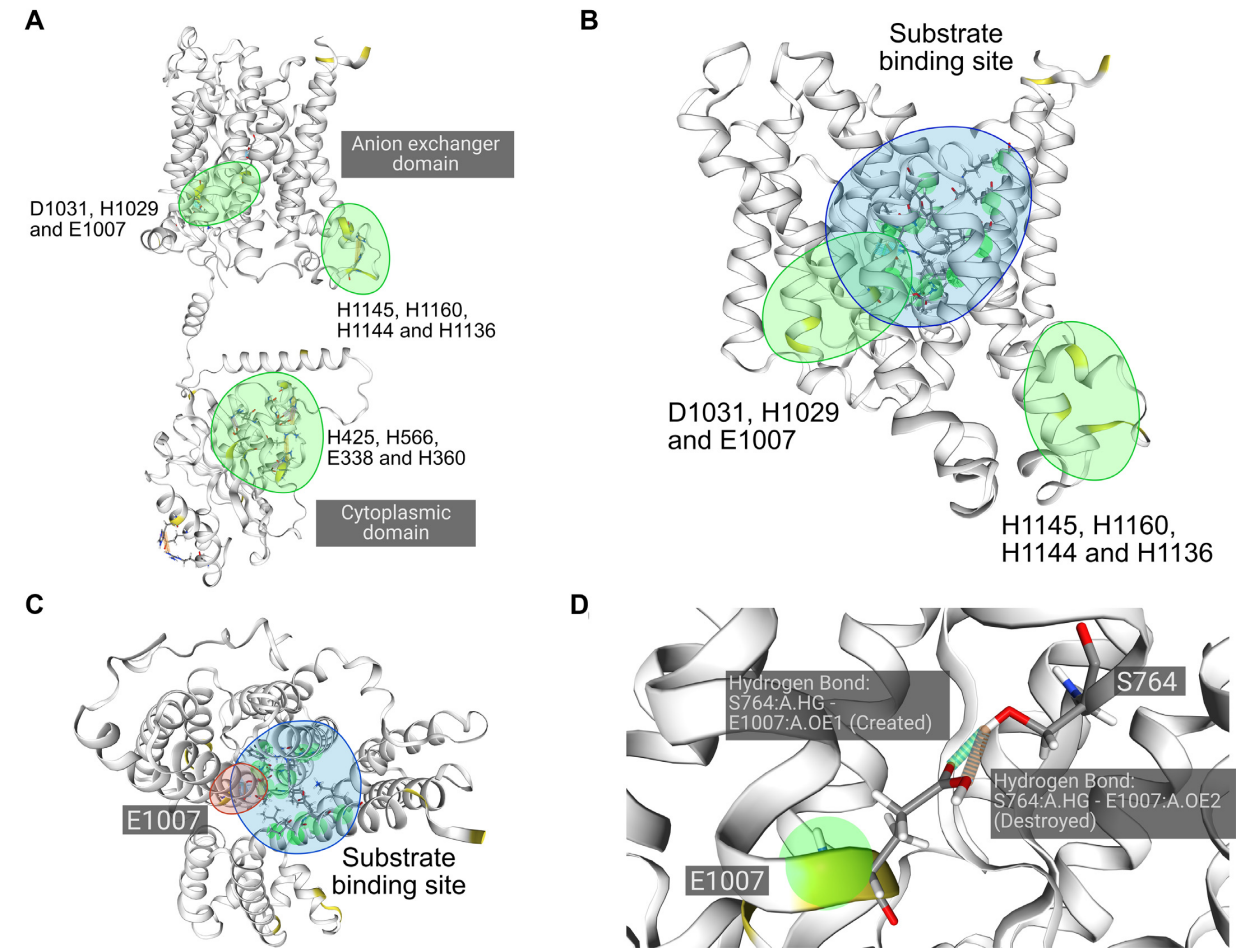
Structure-based patcHwork analysis using a model structure of mAE2 at pH values of 5.5 and 7.5. (**A**) The three pH-sensitive patches identified are highlighted in green. (**B**) Both patches (highlighted in green) of the anion exchanger domain are shown with the mAE2 substrate binding site (highlighted in blue; for details of the residues, see Supplementary Data). (**C**) Top view of the substrate binding site and the differentially charged residue Glu1007 closest to it. (**D**) Shifts in the hydrogen bond between Glu1007 and Ser764, the latter being part of the substrate binding site.

Using patcHwork, it is possible to group pH-sensitive residues into three patches (Figure 3A), resulting in a broader approach compared to using histidine-only information with standard p*K*_a_ values. Looking at the structure, we can speculate that a pH-sensitive region influencing substrate binding is more likely to be found within the anion exchanger domain itself, close to the two substrate binding sites [assuming that these are conserved with the sites found in hAE1 (41); see Supplementary Data] rather than within the cytoplasmic domain. In this case, the patch constituted by Asp1031–His1029–Glu1007 is the most likely pH-sensitive region of mAE2 (Figure 3B). We conclude that, being the closest to the residues involved in substrate binding, residue Glu1007 is the prime candidate to mutate and experimentally test (Figure 3C). Furthermore, as Glu1007 becomes protonated, a shift in the hydrogen bond with Ser764, belonging to the substrate binding pocket, could hint at a restructuring of the binding pocket allowing for a different substrate such as SO_4_^2−^ to bind (Figure 3D). Importantly, our analysis is also in line with an alternative mechanism that was previously proposed, whereby the negatively charged glutamic acid inhibits sulfate binding through ionic repulsion, which ceases when the glutamic acid is protonated (42).

## CONCLUSIONS

patcHwork is a novel, easy-to-use web server that offers users the possibility to perform high-throughput pH sensitivity analysis of protein sequences and structures.

A limitation of patcHwork is that it does not capture pH-dependent structural dynamics that can occur upon pH shift. This can be achieved via molecular dynamics simulations at constant pH (43) comparing the results obtained at different pH values. However, such approaches are computationally demanding and require expertise. On a more intuitive and accessible level, patcHwork allows users to nonetheless predict potential structural rearrangements upon evaluation of gain or loss of noncovalent bonds caused by pH shift.

We believe patcHwork will be an invaluable tool supporting research and teaching, deepening our mechanistic understanding of how pH impacts protein function.

## DATA AVAILABILITY

The web server is freely available at https://patchwork.biologie.uni-freiburg.de.

## Supplementary Material

gkac252_Supplemental_File
